# Bio-Inspired Micro- and Nanorobotics Driven by Magnetic Field

**DOI:** 10.3390/ma15217781

**Published:** 2022-11-04

**Authors:** Anton V. Chesnitskiy, Alexey E. Gayduk, Vladimir A. Seleznev, Victor Ya Prinz

**Affiliations:** Rzhanov Institute of Semiconductor Physics, Russian Academy of Sciences, Siberian Branch, 630090 Novosibirsk, Russia

**Keywords:** magnetic micro-/nanorobots, bio-inspired robots, magnetic field, biomimetic materials, soft robotics, magnetic composite

## Abstract

In recent years, there has been explosive growth in the number of investigations devoted to the development and study of biomimetic micro- and nanorobots. The present review is dedicated to novel bioinspired magnetic micro- and nanodevices that can be remotely controlled by an external magnetic field. This approach to actuate micro- and nanorobots is non-invasive and absolutely harmless for living organisms in vivo and cell microsurgery, and is very promising for medicine in the near future. Particular attention has been paid to the latest advances in the rapidly developing field of designing polymer-based flexible and rigid magnetic composites and fabricating structures inspired by living micro-objects and organisms. The physical principles underlying the functioning of hybrid bio-inspired magnetic miniature robots, sensors, and actuators are considered in this review, and key practical applications and challenges are analyzed as well.

## 1. Introduction

The development of micro- and nanorobots is a promising field of research for solving biomedical problems, targeted drug delivery, environment detoxification, sensor and actuator design, etc. [1,2,3,4,5,6,7,8]. Acting in a living organism for treatment, imaging, cell transplantation, and hyperthermia tasks imposes additional restrictions on the design. Their dimensions should be small enough (a few millimeters or less) to pass through the biological pathways of the body in a viscous environment, should have wireless control, and should be made of non-toxic and biodegradable materials [9].

**Figure 1:**
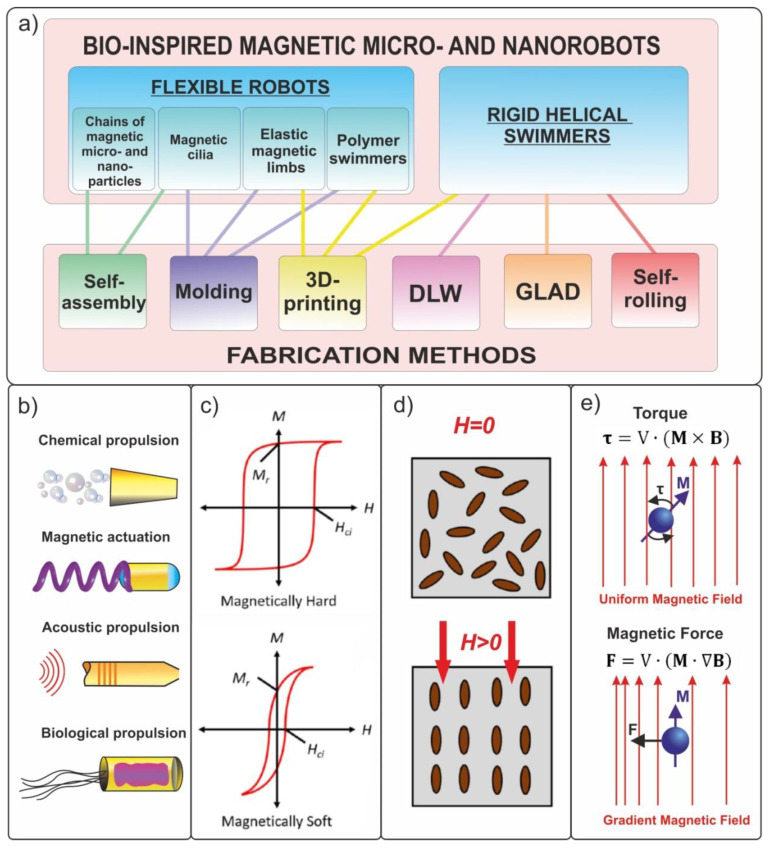
(**a**) Approaches to the fabrication and control of magnetic microrobots. (**b**) Methods for actuating biomimetic micro- and nanorobots. (**c**) Dependence of magnetization on the external magnetic field for hard and soft magnetics. (**d**) Schematic representation of a polymer with magnetic particles cured without and in the presence of a magnetic field. Reproduced from [24]. CC BY 4.0. (**e**) Movement of magnetic object in a uniform and non-uniform magnetic field.

Currently, the suitable architectures of micro- and nanorobots are being actively developed. One of the most promising areas is the development of biomimetic devices (Figure 1a). Over the course of evolution, nontrivial and surprisingly efficient mechanisms for transporting substances inside the body, movement organs, and sensors have been formed by nature. The creation of new materials and devices with biomimetic architectures has already demonstrated the fruitfulness of this concept [10,11]. Modern researchers have to «just» borrow from nature designs of already-existing organisms and micro-objects. The main difficulties of this approach are the high-precision manufacturing of three-dimensional structures, as well as remote control of micro- and nanodevices. The rapid development of 3D micro- and nanostructuring technologies has led to the advent of many methods for the creation of artificial biomimetic structures [12,13,14,15,16]. The development of biocompatible and non-toxic energy sources to ensure the long-term autonomous movement of artificial objects inside living organisms (in vivo) at the temperature of 37 °C remains an important challenge. The following methods of movement of micro- and nanorobots are known from the literature: electromagnetic radiation, ultrasound, chemical reactions, and electric or magnetic fields (Figure 1b) [17,18,19,20,21]. Currently, chemically active components have a short service life due to the rapid consumption of fuel, and they can release toxic substances that are harmful to the body [22,23].

Magnetic fields offer the most promising means for the efficient contactless remote control of micro- and nanorobots movement [24,25,26]. Magnetic methods ensure a sufficiently high speed and stability of movement, allow load transportations, and provide high positioning accuracy [27]. Moreover, the use of weak magnetic fields for the in vivo fuel-free actuation of micro- and nanorobots in a number of medical applications is absolutely safe and offers a minimally invasive solution for living organisms [28,29,30]. The transfer of single or collective biomimetic magnetic micro- and nano-objects [31,32,33] is straightforward to organize using homogeneous, inhomogeneous, gradient, or rotating magnetic fields [34,35,36,37,38,39]. Usually, magnetic control systems are arrays of coils that create magnetic fields of the required magnitude and spatial configuration [40,41,42,43,44].

Physical principles describing the operation of hybrid miniature robots, sensors, and actuators, as well as the practical realization of these devices, have been considered [45,46,47]. In the present study, we consider the main biomimetic magnetic polymer-based flexible and rigid micro- and nanostructures, and their propulsion by an external magnetic field. Particular attention has been paid to the latest advances in the rapidly developing field of magnetic composite design and bioinspired structure fabrication. The bioinspired miniature magnetic structures described in the present review offer highly promising tools for a number of applications, especially biomedical ones [48,49].

The present review is devoted to bioinspired micro- and nanorobots driven by the external magnetic field for tasks of targeted drug delivery and some other therapeutic solutions for oncology, hematology, medical imaging, etc. A comprehensive classification and systematization of the existing types of magnetic robots is carried out, and prospects for their application and further development are identified. The main focus is on the current state of the fabrication issues of these smart biomaterials and devices, as well as the physical basis of their control with the help of external magnetic actuation.

## 2. Approaches to the Fabrication and Control of Magnetic Microrobots

### 2.1. Main Methods of the Prototyping and Fabrication

This chapter briefly discusses various types of soft magnetic composite and the fabrication methods for biomimetic micro- and nanorobots from these materials, and compares the fabricated micro- and nanorobots with known biological microorganisms in terms of size and speed characteristics. The most widely used approach for designing bioinspired magnetic structures is the use of flexible magnetic composites [50,51,52]. Magnetic composites combine ferromagnetic properties while being more convenient from the technological point of view, in comparison with traditional materials, and they are suitable for the formation of devices with complex geometries. The formation of soft magnetic composites [53] is based on the addition of several percent of magnetic micro- or nanoparticles into a polymer matrix [54]. Magnetic fillers can be made from different material types (Figure 1c), including soft magnetics with a weak coercive force (Hc) (Co, Ni, Fe, and NiFe) and hard magnetics with a strong Hc (FeCo, FePt, and FeNdB) and superparamagnetics with zero Hc (Fe_3_O_4_ or Fe_2_O_3_) [55]. The choice of filler is determined by the device interaction with a magnetic field. Soft magnetics have near-zero remanence and are easily magnetized in an external field. Therefore, they are suitable for assembling particle chains. Hard magnetic materials are used in microrobots with a predefined magnetization profile, actuated by an external magnetic field with values less than Hc. Because particles made of hard magnetic materials are tiny permanent magnets, they can be used in biomimetic tactile sensors to detect remanent magnetization. In the absence of an external magnetic field, ferromagnetic particles are uniformly distributed over the volume of the polymer matrix, the structure of which possesses no pronounced anisotropy. The application of a constant magnetic field during the polymerization of polymers and elastomers allows the fabrication of structures with desired magnetic anisotropy and magnetization (Figure 1d) [24,56,57]. Thus, the resultant composite presents an effective magnetic medium which has an average magnetic moment per unit volume equal to that of embedded particles.

An important aspect of the design of magnetic biomimetic structures that perform specific functions [58] is the choice of technology necessary for the manufacture of such structures. Currently, the following methods are most widely used [59]:

1. Fabrication by molding or 3D printing [60,61];
2. Self-assembly of structures from nanoparticles in a magnetic field [62];
3. Rolling of stressed hybrid thin films [63,64,65];
4. Glancing angle deposition (GLAD) [66,67];
5. 3D direct laser writing (DLW) lithography [68].

All of the above methods make it possible to form three-dimensional robots down to the nanoscale; however, they suffer from limitations concerning the complexity of shape and materials. The first four methods make it possible to form objects of a simple shape directly from magnetic composites, such as various protrusions, helices, cylinders, and rings.

The direct laser writing method and the two-photon polymerization technique have no restrictions in terms of the geometry of formed objects. Yet, it is limited by the choice of materials. Ultrashort laser pulses focused on a transparent photocurable polymer locally alter its properties and allow the formation of 3D objects while moving the laser beam or polymer base in space. To form a magnetic layer in this case, it is necessary to additionally use the process of sputtering or depositing materials onto prepared non-magnetic 3D objects [69].

Owing to the wide range of functional magnetic fillers, polymer matrices, and manufacturing technologies, the properties of composite materials can be preset to allow biomimetic robots to perform a number of necessary functions. Magneto-active micro- and nanorobotics open up wide possibilities for the remote manipulation of micro-objects, performing surgical operations and therapy at the cellular level, treating oncological diseases with hyperthermia methods, and restoring the lost functions of living organisms [70,71,72,73].

The sizes and maximum propulsion speeds of biological microorganisms and artificial bioinspired magnetic robots are compared in Table 1. The generalization of the literature data has shown that the speed of magnetic robots movement is usually several times lower than that of their biological counterparts. This circumstance significantly limits the practical use of such robots. Therefore, further improvements in the design and technology for manufacturing magnetic micro- and nanorobots are required.

### 2.2. Magnetic Actuation Methods

Many microorganisms, including sperm and some forms of flagellar bacteria, vibrate and bend in order to swim. Artificially created magnetic robot swimmers are promising objects for solving a number of biomedical problems, including targeted drug delivery in the body [86,87,88], and minimally invasive and cellular microsurgery [23,89,90,91].

Biomimetic swimmers can be precisely moved when controlled by external magnetic fields in biological fluids of organisms [92,93]. In [94,95], a detailed review of the mechanisms for setting magnetically driven micro- and nanorobots in motion was presented. The authors analyzed the key factors affecting the movement of small-scale robots, such as their size and geometry, the type of magnetic control, and environmental conditions. The basic principle of magnetic control is the use of force acting on a magnetized micro- or nanodevice (Figure 1e). The angular momentum in an external magnetic field is given by:

(1) τ=V·(M×B)

where V is the volume of the magnetic object, and M and B are the magnetization vector of the object and external magnetic field, respectively.

Hence, a force

(2) F=V·(M·∇B)

acts on a magnetized object in a magnetic field to turn the direction of its magnetic moment along the gradient of the magnetic field [89,96].

By the type of control, magnetic robots can be divided into two main groups: those driven by a rotating magnetic field and those driven by an oscillating (changing its strength) magnetic field (*f* = 25–100 Hz). Spiral and helical biomimetic micro- and nanorobot swimmers are usually efficiently controlled using weak (B < 10 mT) rotating magnetic fields with an accuracy of up to hundreds of nanometers [90]. Oscillating fields are used to set flexible magnetic structures in motion that imitate the flapping of flagella and cilia, as well as scallop valves [25,97,98].

### 2.3. Hydrodynamic Performance of the Micro-Objects

Microrobots could navigate inside the body, enabling invasion with minimal damage. Biomimetic structures must satisfy a number of requirements to work under biological conditions, such as viscosity, the aspect ratio of the body’s natural pathways sizes, pH, and so on. The requirements for robots must be taken into account at the design stage. When studying the motion of micro-objects in biological fluids, it is important to take into account the ratio of inertial forces to the forces of viscous friction, which is called the Reynolds number [89]:

(3) $$Re=\frac{\upsilon L\rho }{\eta }$$

where υ is the speed of movement; L is the characteristic length of the object; and ρ and η are the density and viscosity of the liquid, respectively.

On the micro- and nanoscale, even for water, we have Re≪1, because the speed of movement and size are small quantities. In order to understand how the movement of microorganisms in water occurs, one can imagine a situation similar to the movement in a viscous resin. For this reason, first of all, the task is posed to develop and optimize the structures of microrobot bodies adapted to efficient movement in a viscous medium. Living nature has found various solutions to this problem, one of which is the use of flagellar actuators with changing wave-like shape.

## 3. Fabrication of Magnetic Micro- and Nanoswimmers

### 3.1. Flexible Polymer Swimmers

**Figure 2:**
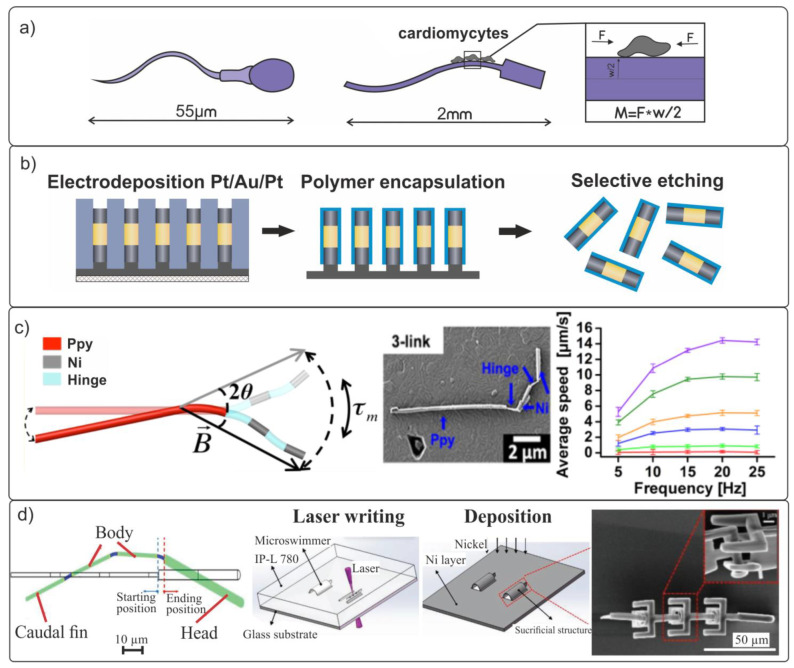
(**a**) Schematic image of biomimetic non-magnetic swimmers. The length of the head and tail of the designed polymer swimmer is ≈400 μm and ≈1500 μm, respectively. The self-propulsion speed reaches ≈10 μm/s. (**b**) The concept of manufacturing magnetic nanorods with a flexible polymer hinge. The image shows the flexible part of the polyelectrolyte shell connecting two Ni/Au/Pt segments. (**c**) Schematic and SEM image of 3-link swimmers [76]. Copyright 2012, American Chemical Society. The graph shows the dependence of the average propulsion speed on the frequency for 3-link swimmers. (**d**) Three-dimensional laser lithography and deposition of a Ni layer for magnetic actuation. The microswimmer made in IP-L 780 polymer using 3D laser lithography and the deposition of a Ni layer for magnetic actuation [77]. The total length of the microswimmer is 120 μm and the length of its magnetic head is ~40 μm.

The first attempt to create an artificial biohybrid flagellar polymer swimmer was undertaken in [99]. The flagellum is propelled by the contraction of cardiomyocytes (muscle cells of the heart) located at its attachment to the swimmer’s body (Figure 2a). The speed of movement of the manufactured non-magnetic swimmers was less than 10% of their body length per second, whose value was much lower than the speed of motion of their biological counterparts. For example, a bovine sperm with a length of 70 μm can swim at a speed of up to 139% of their body length per second (see Table 1). The proposed biohybrid swimmer forms the basis for the development of a new line of research for creating more complex biomimetic micro- and nanorobots.

In a number of works [100,101,102,103], structures of flagellated multilink swimmers controlled by an external magnetic field were proposed and fabricated. In [100], controlled multilink flexible swimmers with 5 μm length of each Ni/Au/Pt magnetic segment were investigated (Figure 2b). In order to imitate the biomimetic bends, the magnetic nanorods were connected together using a 700 nm long flexible hollow polymer cylinder with a thickness controlled with nanoscale accuracy. The flexibility of the entire nanostructure depended on the thickness of the polyelectrolyte layer connecting the magnetic links, and it could be varied depending on the task. Artificial two- or three-link flexible swimmers were capable of exerting bending movements and exhibited a nature-like Brownian movement in water drops.

In [76], multilink nanowire magnetic nanorobots (200 nm in diameter) imitating eukaryotic flagella, under the influence of an oscillating magnetic field, were fabricated and studied (Figure 2c). The tail of the swimmer was prepared using an elastic organic polymer, polypyrrole (Ppy). The magnetic head consisted of rigid nickel (Ni) links connected together by polymer loops. This multilink design proved to be highly efficient for moving in fluids. In the experiment, the magnitude of the external oscillating magnetic field was B = 8 mT. The maximum speed for the three-link swimmer was 14 μm/s (≈0.9 body lengths per second) at a frequency of 20 Hz.

Another promising approach to the fabrication of a biomimetic multilink microswimmer using 3D laser lithography and subsequent local deposition of a 99.99% pure ferromagnetic Ni layer was proposed in [77]. The authors developed a microswimmer design involving four rigid segments connected by joints and requiring no subsequent assembly (Figure 2d). The microswimmer mimics the U-type movement of anguilliform fish, such as eels. The speed of the traveling-wave propulsive motion of the microswimmer in a magnetic field oscillating at a frequency of up to 3 Hz did not exceed ~25 μm/s (≈0.2 body lengths per second).

**Figure 3:**
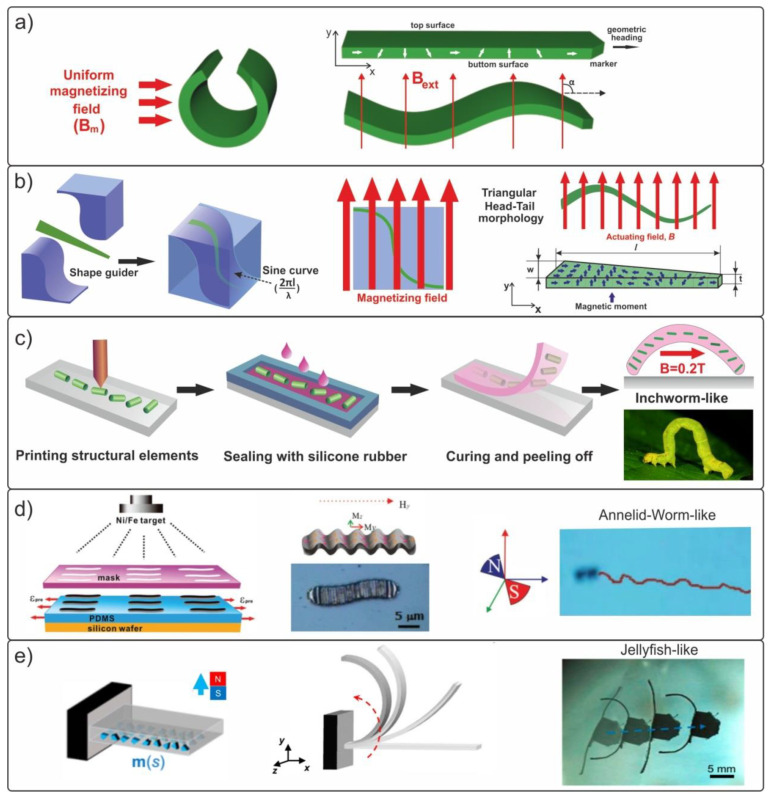
(**a**) Schematic representation of the programming method for the magnetic structure and the resultant sinusoidal magnetization profile of the designed millimeter-sized swimmer. (**b**) Procedure of magnetization along a preset profile in the shape guider and control of a triangular silicone swimmer by an external magnetic field. (**c**) The process of formation of inchworm millimeter-sized magnetic robots using a 3D printer with fused deposition modeling and molding in silicone. (**d**) Schematic representation of the manufacturing process of a magnetic annelid-worm-like microswimmer and its propulsion in a magnetic field [78]. (**e**) Schematic representation of the beam bending under the action of an external magnetic field and the movement of the millimeter-sized swimmer in water, simulating the movement of a jellyfish [108].

The next interesting idea, significantly expanding upon the available possibilities in the design of magnetically controlled bio-inspired micro- and nanorobots, is the formation of elongated structures (rods, stripes) with a preset complex magnetization profile (Figure 3a,b) [78,104,105,106]. The concept of swimming magnetic robots was proposed using an example of structures with a sinusoidal magnetization profile [104,107].

The millimeter-sized artificial swimmer moves due to the internal deformations which arise when a uniform rotating magnetic field is applied. Soft millimeter-sized magnetic swimmers were prepared from a highly flexible elastomer (Ecoflex 00-50, Smooth-on Inc., Macungie, PA, USA) with a density of 1.07 g/cm^3^ and a Young’s modulus of 83 kPa from NdPrFeB unmagnetized hard-magnetic microparticles (diameter d = 5 μm, Magnequench, Central Singapore, Singapore) taken in a mass ratio of 1:1 [104]. Molding with dimensions of 1.5 mm × 4.9 mm × 60 µm was cured from the composite mixture. Then, to set the magnetization shape, the swimmer was rolled into a ring and placed in a uniform magnetic field of 1 T, normal to its axis, as shown in Figure 3a. Following this procedure, the strip in the unrolled state had a sinusoidal magnetization profile with an amplitude of M = 48 kA/m along the body of the swimmer:

(4) $$M\left(x\right)=\mathrm{Mcos}\left(\frac{2\pi x}{\lambda }\right)i+\mathrm{Msin}\left(\frac{2\pi x}{\lambda }\right)j$$

where λ is the wavelength of the sinusoidal profile, and **i** and **j** are the unit vectors in the x and y directions, respectively. The authors paid significant attention to the mathematical model of the motion of a flexible magnetic swimmer in an external magnetic field. It was shown that, in rotating magnetic fields, a sinusoidal magnetization profile leads to the generation of a strain wave propagating along the swimmer body. This wave pushes the magnetized strip and causes it to move in a swimming manner. In the experiment, the highly efficient controlled propulsion of the robot in water at a speed of 50 mm/s (10 body lengths per second) was demonstrated, which is comparable to that of biological objects.

Later in 2020, a bio-inspired millimeter-scale silicone structure with a similar sinusoidal magnetization profile was realized in [104]. A triangular magnetic swimmer with a body size of 2 mm × 9.5 mm × 80 µm was manufactured by the traditional molding method from a composite silicone polymer with NdFeB microparticles taken in 1:1 ratio, followed by magnetization in the mold in a magnetic field of 650 mT (Figure 3b). This method of setting the orientation of the magnetization along a sinusoidal curve demonstrates the self-propulsion at a speed of only 4.5 mm/s (~2 body lengths per second) in a uniform oscillating magnetic field of 12 mT due to the generation of wave-like deformations of the body.

Qi et al. [106] used an unconventional approach to fabricate a biomimetic inchworm silicone robot using 3D printing of structural magnetic elements from a composite of polylactic acid (PLA) and carbonyl iron particles (CIPs, 1–8 μm, BASF, Ludwigshafe, Germany). As a result, it was shown that a flexible silicone robot with 3D-printed soft magnetic fibers was capable of performing fast, reversible, and stable body deformations in a uniform magnetic field, as shown in Figure 3c.

Significant progress has been made in the development of millimeter-sized worm-shaped robots [109,110,111,112]. It was shown that flexible biomimetic robots with magnetic segments can deform part of their body in the O-shape and generate biomimetic creeping movements, including movements in a liquid medium.

In another study using similar approaches [78], annelid-shaped microswimmers with a body length of ~20 µm were fabricated by sputtering a Ni/Fe magnetic layer through a shadow mask onto a pre-deformed elastic polydimethylsiloxane (PDMS) substrate. After the relaxation of the stress in PDMS, a sinusoidal wrinkling profile was formed on the surface (Figure 3d). It was shown that in an external magnetic field of up to 70 mT, the speed for annelid-worm-like microswimmers with a fold period of *λ* = 1.5 µm reached 100 µm/s (~5 body lengths per second).

Lum et al. [108] proposed the concept of bio-inspired millimeter-sized swimmers with two tentacles that have a programmed magnetization profile (Figure 3e). The silicone tentacles were capable of generating rapid swings that mimicked jellyfish movement in water. The swimmers were fabricated using NdFeB magnetic particles (d = 5 µm, Magnequench, Singapore) embedded in a silicone matrix (Ecoflex 00-10; Smooth-on Inc., Macungie, PA, USA), and they exhibited a low speed of movement of 1.8 mm/s. The volume fraction of magnetic microparticles in the Ecoflex polymer was 0.15:1. The proposed shapes and designs of biomimetic millimeter robots with programmed magnetization profiles have great practical potential, and, in the near future, they can be scaled down to the nanoscale.

**Figure 4:**
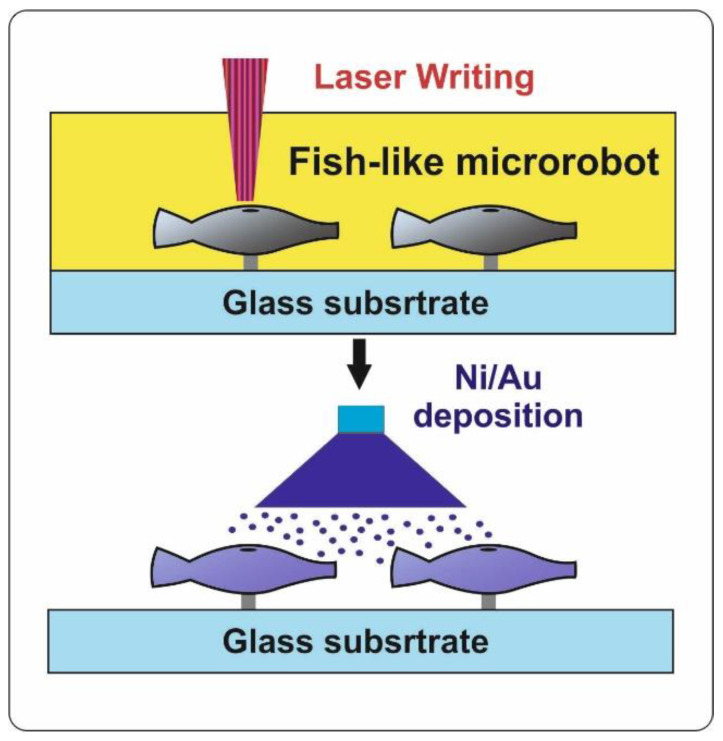
Schematic representation of the manufacturing process of dual-driven biomimetic microrobots.

In a recent publication, researchers proposed a unique dual-driven biomimetic structure propelled by infrared light and magnetic field [75]. The microfish body was fabricated from biocompatible SU-8 photoresist using rapid 3D printing technology (DLW) (Figure 4). Then, the deposition of functional Ni and Au nanoparticles was performed for magnetic and optical control, respectively. When controlled using a magnetic field gradient, the average translational velocity of the artificial microfish reached a value of 220 µm/s (2.4 body lengths per second) in deionized water. The micromovement of the robot, due to the heating of its body during the absorption of infrared light and the occurrence of thermal convection flow, was also demonstrated in this work. The ability to move at high speeds under magnetic control and with precise movement under optical control in microfluidic channels under harsh fluid conditions has been demonstrated. The proposed structure with dual control opens up various opportunities for the development of intelligent micromotors that can reconfigure their movement mode depending on the problem being solved and environmental conditions.

### 3.2. Chains of Magnetic Micro- and Nanoparticles

It is known that living organisms are sensitive to magnetic fields and, moreover, the geomagnetic field has a huge impact on the migration and movement of animals. The remarkable ability of animals (e.g., termites, bees, and fruit flies) to detect magnetic fields is called magnetoreception, and in bacteria this ability is called magnetoaxis [113,114]. This process occurs because of the presence of magnetosomes in the cells. Magnetosomes are nanocrystallites (~100 nm) of magnetite (Fe_3_O_4_) and greigite (Fe_3_S_4_) covered with a protein membrane [115]. Inside the cell, magnetosomes are arranged in a chain and are fastened together. Such chains are cellular sensors that sense the direction and gradients of the earth’s geomagnetic field. Researchers have shown how to self-assemble 3D-architectured systems from nano-objects for deep tissue treatment. Designed structures can be brought into the therapy area and assembled on site into the desired shape using external fields for microsurgical purposes.

**Figure 5:**
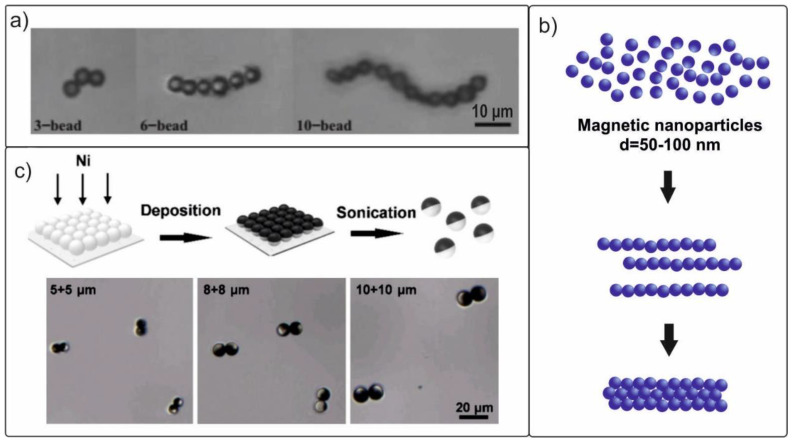
(**a**) Photos of microswimmers of various lengths collected from chains of microparticles in a magnetic field. Reproduced from [83]. CC BY 4.0. (**b**) Three-stage formation of micro- and nanoswimmers based on iron oxide nanoparticles. (**c**) Design and manufacturing steps of magnetic microdimer swimmers and an optical image of Janus microdimers after magnetization. Reproduced from [85]. CC BY 4.0.

Artificially created self-assembling magnetic chains used for movement in biological fluids have been investigated in a number of studies [62,83,84,116]. In the research [83], external conditions for the occurrence of reversible processes of assembly and disassembly of microparticles (4.35-µm particle diameter, Spherotech Inc., Lake Forest, IL, USA) in chains of various lengths (Figure 5a) were identified. The authors developed an algorithm for the formation, using a rotating magnetic field, of chains, not only in the form of a straight line, but also with a bend. It was shown that at a field frequency of 6 Hz, the average speed of a swimmer formed using 13 microparticles reached ~18 µm/s. In another study [62], the same research team formed self-assembling nanoswimmers (Figure 5b) from nanoparticles 50–100 nm in diameter (iron (II, III) oxide, Sigma-Aldrich, Taufkirchen, Germany).

Later, Yu et al. [84] reported the high efficiency of the movement of Janus microdimers in a rotating magnetic field. These microdimers were composed of two Ni/SiO_2_ microspheres held together by a magnetic force. Microparticles with sizes of 5, 8, and 10 µm were used in the experiment (Figure 5c). For the microdimers to move along the *X* axis using a system of Helmholtz coils, a circularly polarized rotating magnetic field H(t) = H0[cos(ωt)ex + sin(ωt)ez] was applied in the XZ plane.

The propulsion of microdimers sized 5 + 5 µm at a maximum speed of 133 µm/s (~13 body lengths per second) in a 32 Hz magnetic field of 5 mT was demonstrated. A change in speed was easily achieved by varying the strength and frequency of the magnetic field. However, a further increase in frequency led to a decrease in the propulsion speed. The proposed original Janus microdimer swimmers open up new opportunities for the targeted destruction of cancer cells and biomedical operations at the nanoscale.

### 3.3. Rigid Helical Swimmers

**Figure 6:**
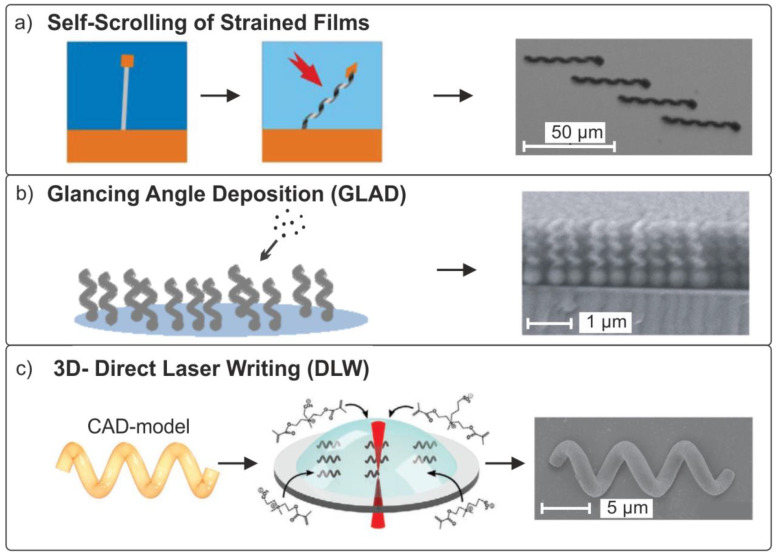
Fabrication methods of magnetic helical swimmers. (**a**) A method based on the self-rolling of a three-layer strained film with a nickel magnetic head [63]. Copyright 2009, American Chemical Society. (**b**) Glancing angle deposition (GLAD) method [120]. Copyright 2009, American Chemical Society. (**c**) Three-dimensional direct laser lithography (DLW) [117]. John Wiley & Sons (Hoboken, NJ, USA). Copyright © 2020 WILEY-VCH Verlag GmbH & Co. KGaA, Weinheim, Germany.

**Figure 7:**
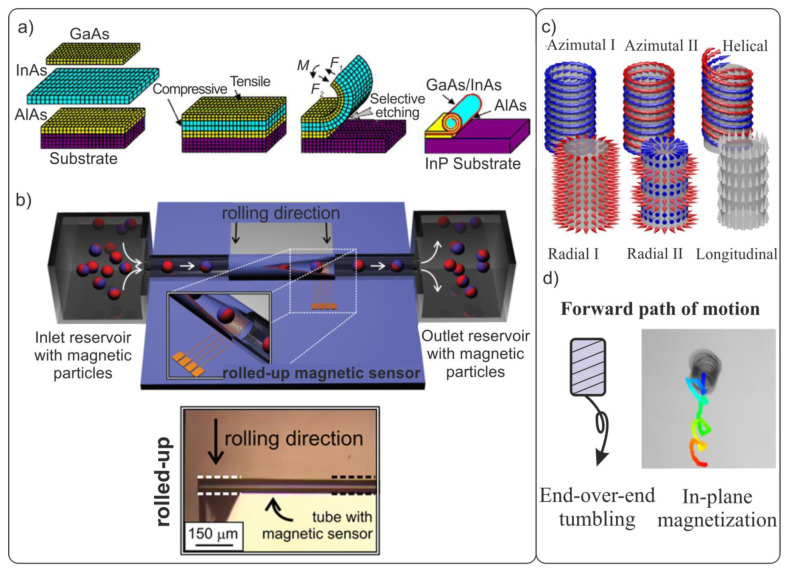
(**a**) Stages in the formation of arrays of rolled-up semiconductor and polymer 3D shells. The rolling of strained films in a tube scroll occurs after their separation from the substrate by selective liquid etching [13]. (**b**) Schematic representation of a rolled-up multilayer GMR sensor for the in-flow detection of magnetic objects inside a microfluidic channel [124]. Copyright 2011, American Chemical Society. (**c**) Domain structure in bent cylindrical films. Reproduced from [121], © IOP Publishing. Ltd. All rights reserved. Top row: circular magnetization without a longitudinal component (azimuthal) and with a longitudinal component (helical). Bottom row: radial and longitudinal magnetization. (**d**) Tracking the trajectory in magnetic field of a rolled-up magnetic coil with in-plane magnetization [125].

In wildlife prokaryotic bacteria, rotating flagella is used to move in the fluids. This principle of microorganism propulsion is based on the transformation of rotational movement into a linear movement. In recent years, a large amount of research has been conducted on helical swimmers and their fabrication methods. The figure below shows the key techniques for the formation of biomimetic helical structures using group technology (Figure 6a,b) and rapid prototyping (Figure 6c) [63,89,108,117]. The first method [65] uses the physical mechanisms of elastic deformation in thin strained films, allowing the films to bend and roll up into a scroll tube or helix when released from bonding with a massive substrate (Figure 7a). This method allows the formation of periodic arrays of high-precision three-dimensional structures with sizes as small as 2 nm [118,119].

Later, the magnetic properties of bent thin magnetized films were described [121]. The authors considered fundamental issues concerning the influence of the bending of magnetic films on the topology of their magnetization (Figure 7c). From a practical point of view, the predefined magnetic anisotropy of bent films makes it possible to control the 3D movement and rotation of micro-objects in an external magnetic field for several biological and microfluidic applications (Figure 7d). Moreover, the considered technology of rolling strained films in a scroll is also widely used for the fabrication of tubular GMR and magnetic Hall sensors (Figure 7b). This allows the tracking and reading of magnetic markers inside rolled microfluidic channels [122,123,124].

**Figure 8:**
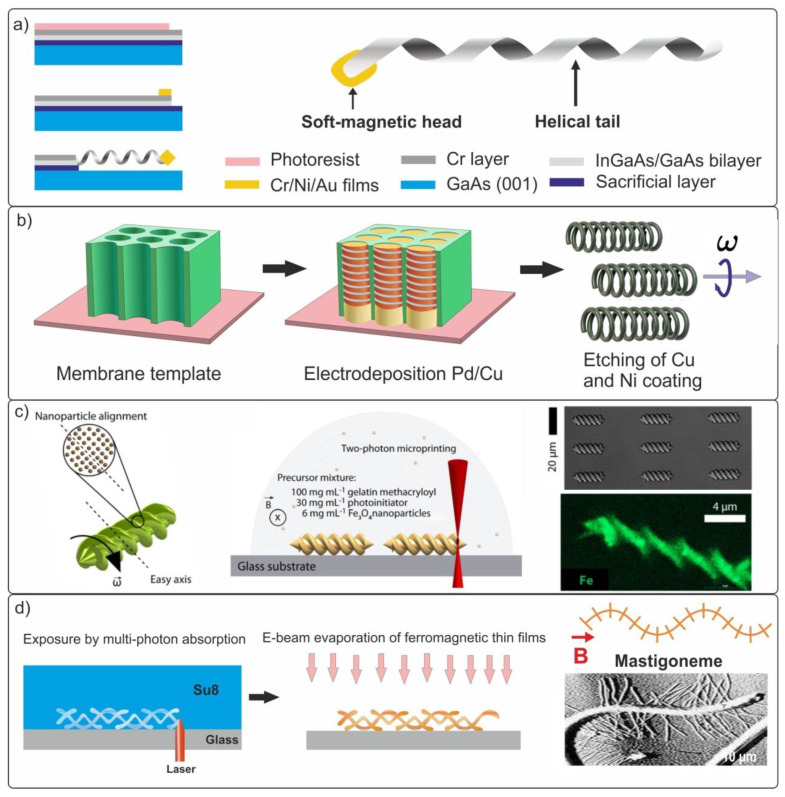
(**a**) The main manufacturing stages of the helical semiconductor InGaAs/GaAs/Cr swimmer. (**b**) Schematic representation of a template-based manufacturing process of helical nanoswimmers of 800 nm in length. (**c**) Manufacturing process of a 3D magnetic biodegradable swimmer using the two-photon polymerization method. Images of hydrogel microcoils obtained using optical microscopy and energy-dispersive X-ray spectroscopy [80]. (**d**) Photo of Mastigonemes of Ochromonas and the manufacturing stages of a biomimetic rigid microswimmer.

Zhang et al. [63,81] proposed a technique for fabricating helical semiconductor swimmers based on the self-rolling of a multilayer strained InGaAs/GaAs/Cr film. It is important to note that this method of forming arrays of biomimetic microswimmers is compatible with traditional planar semiconductor technology. Figure 8a comprehensively describes the main stages in the formation of helical microswimmers with a soft Cr/Ni/Au magnetic head of square shape with dimensions of 4.5 × 4.5 × 0.2 µm.

In a weak magnetic field (B < 2 mT), the propulsion speed of the left-handed microswimmer with a body length of l = 38 µm reaches 1.1 µm/s and 1.8 µm/s in the forward and opposite directions, respectively. The authors demonstrated the possibility of moving loads in deionized water in the form of two polystyrene microspheres with a diameter of 6 µm (Polysciences Inc., Warrington, PA, USA). Another inexpensive template electrosynthesis method for the formation of nanohelical swimmers was proposed to efficiently solve the problems of targeted drug delivery and biopsy [79]. The mechanism was based on the electrodeposition of Pd/Cu nanorods into a porous polycarbonate nanomembrane (PC, Whatman, NJ, USA), followed by the removal of Cu and the deposition of a Ni magnetic layer (Figure 7b).

As a result, nanohelices with extremely small dimensions were obtained (the diameters of the nanohelices were d = 100, 200, and 400 nm). The propulsion speed of nanoswimmers with a body length of 3 µm (d = 400 nm) in a rotating magnetic field was 15 µm/s, which corresponds to a relative speed of approximately 5 body lengths per second. The obtained speed value is one of the best in comparison with helical micro- and nanoswimmers fabricated using other methods, such as self-scrolling and GLAD.

However, the above-described swimmers have a significant disadvantage in terms of biocompatibility and biosafety after completing tasks within a living organism. Later, in several studies [73,80], methods for the 3D printing of biodegradable swimmers from safe materials containing Fe3O4 nanoparticles were proposed. Manufactured by a two-photon polymerization process from a biocompatible hydrogel matrix, magnetic microswimmers completely degrade into non-toxic soluble products within 118 h at 37 °C (Figure 8c) [80]. The propulsion speed of microswimmers with a body length of 20 μm and a diameter of 6 μm in a rotating field was ~3.5 µm/s. The helical microswimmers reported in [73] exhibited much better characteristics. The propulsion speed of swimmers with a body length of 120 μm reached 114 µm/s and the composite hydrogel with Fe_3_O_4_ nanoparticles completely decomposed within 30 h.

The authors of [126] developed a design of biomimetic rigid helical microswimmers inspired by the mastigonemes cilia from the wild world. Mastigonemes usually cover the tails of flagellar bacteria in a perpendicular direction, as shown in Figure 8d, and it is these organs that define the reverse direction of movement. Microswimmers with extraordinary body geometry were fabricated from a negative photoresist (SU-8 50, MicroChem, Round Rock, TX, USA) using three-dimensional lithography, and their surfaces were then covered with a thin ferromagnetic film (100 nm of Ni and 5 nm of Ti) using electrodeposition. Experimentally, it was shown that an increase in the specific number of mastigonemes per turn of the helix led to a decrease in the swimming velocity in the forward direction, and at a certain moment, it caused reverse propulsion of the artificial microswimmer, similar to the movement observed in nature.

**Figure 9:**
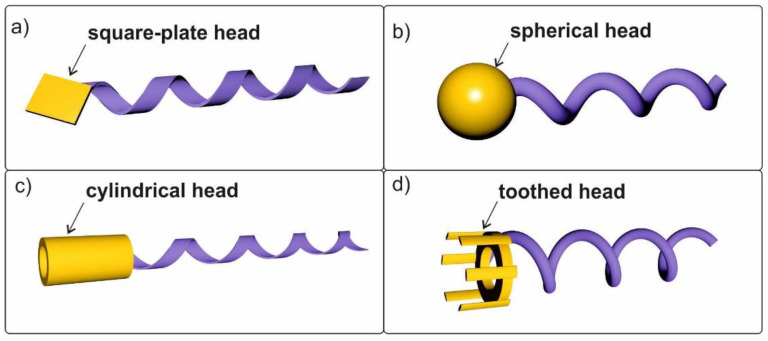
Helical swimmers with various magnetic heads: (**a**) square-plate-head swimmer; (**b**) spherical-head swimmer (GLAD); (**c**) cylindrical-head swimmer; (**d**) toothed-head swimmer.

A comparative experimental and simulation study of rigid helical swimmers with different magnetic head shapes was reported in [127]. The main types of swimmers are shown in Figure 9 [81,120,128,129]. The authors paid special attention to the study of the dependence of the rotation frequency of helical swimmers on different shapes of magnetic heads. The shape of the magnetic head of the swimmers was shown to be a factor affecting their propulsion in low-Reynolds-number liquids. The helical magnetic swimmer without a head had the highest cut-off frequency, because it was the head that generated additional braking torque.

Shape memory materials, which are promising for the manufacture of helical biomimetic microrobots, can be reversibly deformed under various external influences (temperature, electric and magnetic fields, pH, and moisture) [130,131,132]. Usually, this class includes alloys of several metals (Cu, Co, Ni, Au, Zn, and Cd). Moreover, there are also shape memory polymers, and some of them are biodegradable [133]. Coronary stents with a memory effect are already used in endovascular surgery in vessels as reinforcing structures and usually have dimensions of a few millimeters. Microscale memory structures like these can expand the functionality of magnetic swimmers. They will allow the shape of the swimmers to be changed (expanding the diameter of the helix or its pitch) during the tasks, and the movement is carried out using an external magnetic field. For example, in the work [134], programmable shape microswimmers implemented by winding technology are proposed. The maximum speed proved to be 6 body lengths per second. With the help of shape alloy memory, it is possible to deliver a biomaterial or drug into a cell using a helix swimmer and release it owing to external influence. It will allow conduct operations in difficult-to-reach anatomical spaces, small vessels, and brains [135,136].

## 4. Synthesis of Magnetic Biohybrid Robots

Miniature biohybrid robots are functional combinations of living cellular materials and inorganic magnetic structures. Currently, two approaches are known for forming biohybrid robots. The first approach is based on the use of magnetic structures for moving immobilized or weakly mobile cells, or for the delivery of a useful load in the form of a biomaterial. The second approach is based on the use of additional biological camouflage coatings on magnetic micro- and nanostructures to increase their biocompatibility with patients.

**Figure 10:**
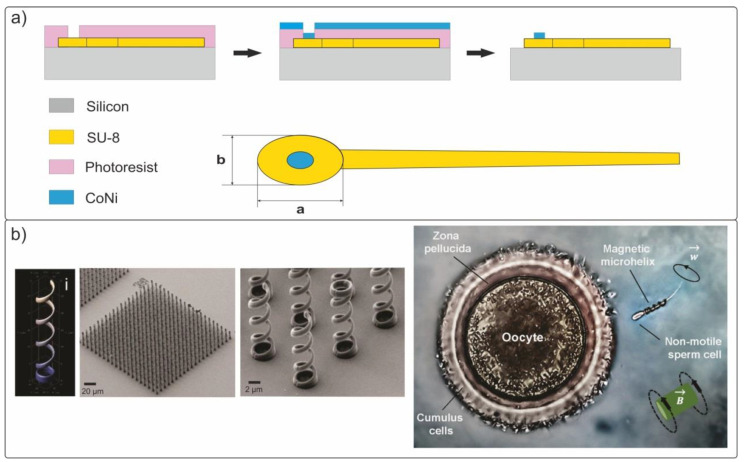
(**a**) Schematic structure of a biomimetic spermatozoon of 322-µm length. The dimensions of the ellipsoidal head are a ≈ 42 µm and b ≈ 28 µm. (**b**) SEM photos of four-turn microhelices and the process of transporting a sperm cell captured by a magnetic microhelices [138]. Copyright 2016, American Chemical Society.

Previously, completely inorganic magnetic (Co_80_Ni_20_, d = 200 nm) artificial spermatozoa with a flexible polymer (SU-8) tail were proposed (Figure 10a) [74]. The maximum propulsion speed of the fabricated samples with a body length of 322 µm reached 158 µm/s at a magnetic field frequency of B = 5 mT.

Later, the comparable concept of a hybrid micromotor for transporting immobile or completely immobilized spermatozoa was presented [137,138]. Individual magnetic microhelices served as motors for transporting immobile living spermatozoa in fluid channels. Helical polymer micromotors (Figure 10b) were fabricated using direct laser lithography and were coated with a soft magnetic NiTi bilayer, as described in detail in [139].

The movement of the microhelices in the liquid proceeded in a rotating magnetic field. The experiments were carried out under simulating physiological conditions inside fluid channels prepared from glass and thin laboratory films (Parafilm^®^, Switzerland). The photo (Figure 10b) shows the process of egg fertilization with motionless sperm owing to sperm delivery by remotely controlled magnetic microhelices. The authors had managed to isolate single spermatozoa, capture them in liquid, and transport them in an external magnetic field created by a Helmholtz coil system. The speed of the controlled three-dimensional motion of spermatozoa at temperatures 20–40 °C was comparable to the speed of living spermatozoa (10–70 µm/s). In this work, the authors investigated the influence of the geometric parameters of the helix, such as the pitch, length, and number of turns, on the characteristics of the motion. It was experimentally shown that a helix with four turns usually developed higher speeds than a helix with three turns, for instance ~55 m/s compared to ~35 m/s, respectively, at a frequency of 50 Hz in a physiological environment. This work demonstrates the high reproducibility of the shape and characteristics of artificial micromotors owing to the accurate choice of lithography and magnetic coating modes. Although there remain some obstacles on the road to artificial insemination, significant and important steps have been taken to achieve successful fertilization with motile artificially motorized sperm.

In another study [140], living subspecies of the cyanobacterium *Spirulina* (*S. platensis*) were chosen to manufacture a magnetic biorobot. These are ready-made helical microstructures with a certain pitch, diameter, helical angle, and chirality. All these characteristics are sensitive to the living conditions of *Spirulina*, and they can be adjusted in the laboratory during cultivation. This allows for the mass production of specimens with optimum geometric parameters as a biological matrix for manufacturing a biohybrid robot. In addition, the length of the helix can be further adjusted, as required by mechanical cutting during the ultrasonic treatment.

**Figure 11:**
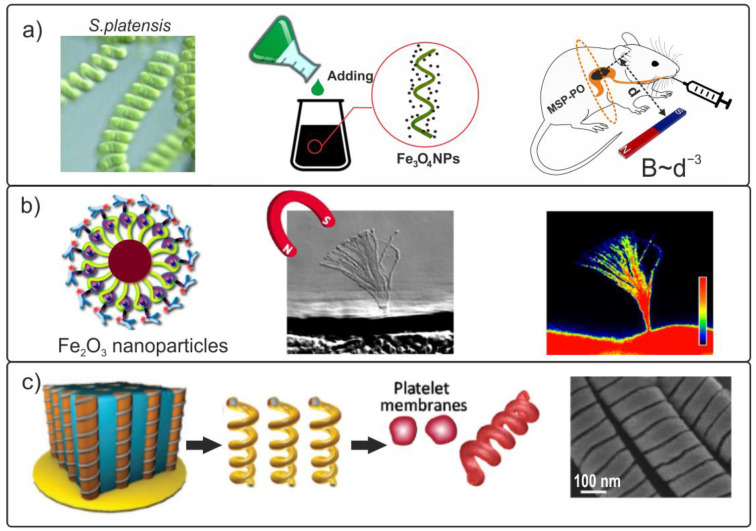
(**a**) Covering of S. platensis with magnetite and the MRI study of biohybrid microrobots inside a rodent. (**b**) Demonstration of Fe_3_O_4_-coated biohybrid magnetic cilia for treating the polycystic kidney disease [143]. Copyright 2019, American Chemical Society. (**c**) Manufacturing process of magnetic helical nanomotors coated with platelet membranes, and SEM photo of fabricated bare helical nanomotors [144].

Helix-shaped magnetic biohybrid robots were manufactured by integrating biological materials with low-cytotoxicity magnetite nanoparticles [141]. Biorobots were formed from *Spirulina* microalgae coated with superparamagnetic magnetite particles (Fe_3_O_4_) from an aqueous suspension (Figure 11a). The authors had chosen magnetite nanoparticles due to their low toxicity to cells of living organisms, the possibility of preparing stable suspensions from such nanoparticles (without agglomeration), and the high contrast produced by such nanoparticles in magnetic resonance imaging [142].

The fabricated magnetic biohybrid robots were set in motion using a Helmholtz coil system. Because the particles were superparamagnetic, they had no remanent magnetization. This meant that they would not collect in agglomerates that are dangerous to the body and cause embolism. In this study, experiments were performed based on the in vivo fluorescence imaging of subcutaneous tissues and the intraperitoneal cavity of nude mice. It was demonstrated that the intense emission of red light from samples of magnetite-coated *Spirulina* (MCS) could easily be observed inside mice upon excitation with green light without the addition of any fluorescent markers. However, the capability of green light to penetrate tissues is limited to a depth of several centimeters, which does not meet the requirements of many clinical applications. Fluorescence imaging stops working in an organ as deep as the stomach. In this case, the authors used an alternative highly efficient imaging technique, known as magnetic resonance imaging (MRI). Figure 11a shows the results of MRI studies. It can be observed that the image contrast had distinct boundaries. The movement of magnetic biohybrid robots in a rotating external magnetic field created by a permanent magnet on a special manipulator was registered.

Pala et al. [143] proposed an innovative approach to the therapy of a genetic disease called polycystic kidney disease (PKD). Sensory cilia were located on the side of the renal tubule lumen in the epithelium cells of renal tubules. As a result of the disease, cilia lose their mobility and become insensitive to urine flow. Cilia-targeted magnetic Fe_2_O_3_ nanoparticles can be used to remotely control the motion of immobile cilia using a magnetic field, as shown in Figure 11b. It has been demonstrated that the use of magnetic nanoparticles is more efficient than the use of short-acting drugs.

Another approach to creating a surface biocoating of a biomimetic nanorobot was described in [136]. Inorganic paramagnetic Pd nanocoils with a diameter of 400 nm were coated with the plasma membrane of human platelets (Figure 11c). As a result, biomimetic nanomotors disguised themselves as human platelets and demonstrated efficient movement in the blood for a long period of time. Such nanomotors offer great potential for a variety of biomedical and bioprotective applications.

**Figure 12:**
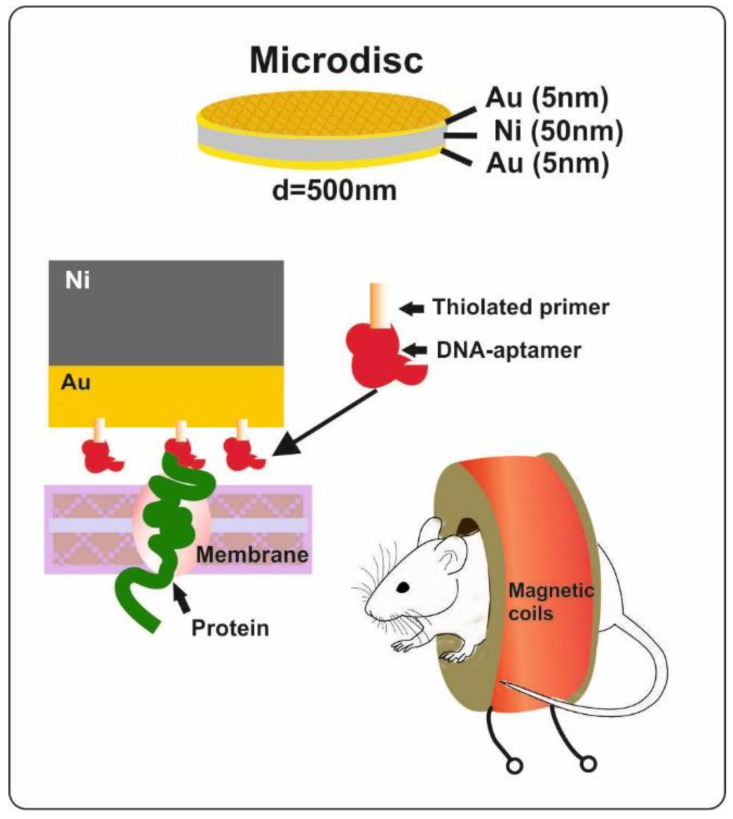
Au-Ni-Au magnetic microdisks with functional DNA bioaptamers used to destroy mouse cancer cells.

Today, numerous magnetic nano- and microstructures are widely used for the magnetomechanical noninvasive destruction of oncological cells [145]. Paper [69] describes Au-coated nickel magnetic disks, 500 nm in diameter, fabricated using nanoimprint lithography (Figure 12). The nanodisks had a magnetic moment perpendicular to the disk plane that prevented them from sticking together. For selective action on carcinoma cells, special functional aptamers were attached to the Au surface of the nanodisks, which ensured that the disk was attached to the cancer cell membrane only. Under the influence of an alternating magnetic field, the mechanical oscillation of the microdisk leads to the cell destruction. This work demonstrates an efficient therapy for mammary adenocarcinoma in mice using a 100 Hz alternating magnetic field of 10 mT applied for 10 min.

Thus, the creation of biohybrid magnetic micro- and nanorobots is a promising research area, along which considerable progress can be expected in the near future, especially in the field of biomedical applications.

## 5. Bioinspired Elastic Magnetic Limbs

Legged animals usually show excellent adaptability to complex terrain, and they have access to almost 100% of the Earth’s surface. The legs and feet are the result of a billion-year evolutionary process. Limbs of this type are found as equally often in land animals (ants, dogs, cheetahs, etc.) and marine animals (octopus, starfish), and they can be used to implement the movement of microrobots.

Researchers have studied the structure of the feet of many living organisms and proposed their own design of flexible many-legged robots [146,147].

**Figure 13:**
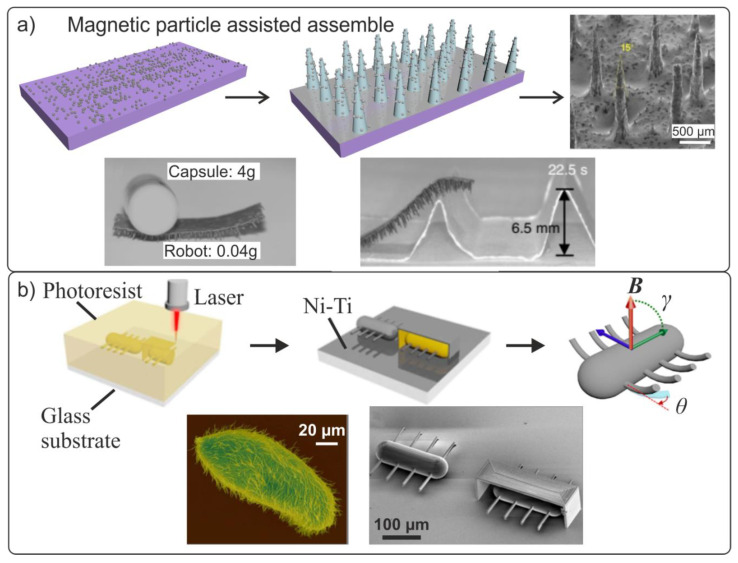
(**a**) Manufacturing stages of a magnetic biomimetic robot and its SEM image. Demonstration of the microrobot’s movement under harsh environmental conditions: (1) movement with a load 100 times exceeding the weight of the robot; (2) overcoming obstacles. Reproduced from [146]. CC BY 4.0. (**b**) Manufacturing stages of the MEMS biomimetic three-dimensional ciliated microrobot and SEM photos of the ciliate microorganism *Paramecium caudatum* and the artificial magnetic microrobot. Reproduced from [82]. CC BY 4.0.

Figure 13a schematically shows the main stages of the magnetic particle-assisted molding approach for magnetic robots with a tapered feet structure [146]. This design provides better adaptability to various environmental conditions, and the ability to function under both dry and wet conditions.

At the first stage, a mixture of polydimethylsiloxane prepolymer with Fe magnetic microparticles was prepared, from which a homogeneous 150 µm thick coating was formed on the substrate. Tapered feet with a height of 650 µm and pitch of 600 µm were pulled out from the liquid coating under the action of a magnetic field. This geometric shape was obtained via solidification in the presence of an external magnetic field in a convection oven. The created biomimetic robot was controlled by an external magnetic field whose strength was varied from 0 to 200 mT. Depending on the configuration and orientation of the applied magnetic field, two types of motion of the artificial foot were demonstrated: (1) a discontinuous flap wave that was observed when the magnetic field was applied in the form of the letter “O” in the y-z plane; (2) continuous inverted-pendulum movement, similar to that of a person, exerted under the influence of a magnetic S-shaped field in the x-y plane. The superior characteristics of the biomimetic elastic magnetic limb ensure an ultrafast travel speed of more than 40 step/s, a super-high lift capacity of up to 100 times the limb’s own weight, and the ability to overcome obstacles at up to 90° angles and 10 times the limb’s own height (Figure 13a).

Other researchers sought to create a fully three-dimensional ciliated microrobot driven by an external magnetic field inspired by the ciliate shoes (*Paramecium caudatum*) (Figure 13b) [82]. The MEMS microrobot was fabricated from a negative photoresist using laser lithography. Subsequently, a biocompatible magnetic Ni/Ti bilayer was sprayed onto the ciliated part of the robot. In this study, the optimum parameters were calculated, such as the length of the cilia, the force and angle of the applied magnetic field, and the force acting on an individual cilium. The translational velocities of the microrobots in various liquids were experimentally investigated. For a microrobot with a body length of l = 220 µm in deionized water (see Figure 13b), and the maximum speed proved to be 340 µm/s (1.55 body lengths per second) in a 60 Hz magnetic field of strength B = 12 mT.

In recent years, the increased interest in bio-inspired flexible multi-legged robots has been driven by the effectiveness of manipulation by external magnetic sources without the need for an on-board power supply. Soft limbs, made from magnetic composite polymers, can be adapted to move efficiently in a liquid medium or vacuum. The use of biocompatible materials for the production of multi-legged biomimetic micro- and nanorobots opens new horizons for a number of minimally invasive medical applications.

## 6. Functional Magnetic Cilia and Tactile Sensors

In nature, arrays of micro- and nanostructures that implement specific functionalities are widely found in plants and animals. The surfaces of many biological organs are covered with various ciliary microstructures. The main roles of the mobile cilia are transportation and movement. For example, in mammals, special cilia cover the surface of the respiratory tract epithelium in order to remove phlegm from the body.

**Figure 14:**
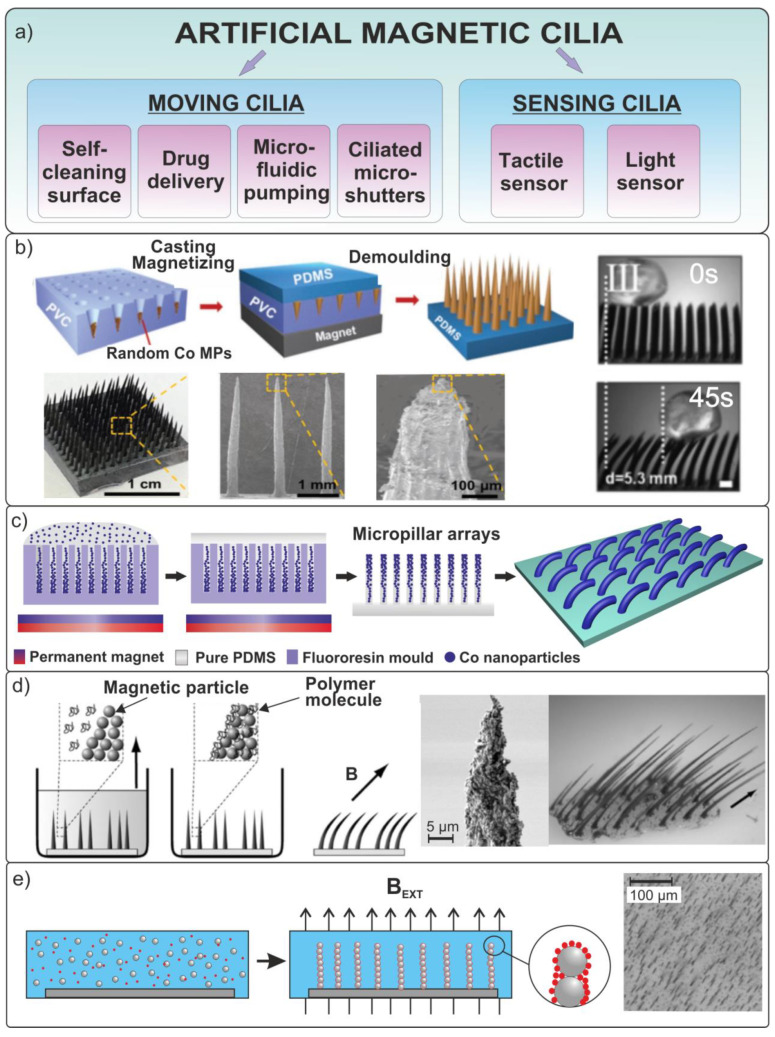
(**a**) Function of biomimetic magnetic cilia. (**b**) Fabrication procedure of magnetically flexible conical arrays. Observation of the movement of a microsphere effected by an array of magnetic cilia in an external magnetic field of B = 0.7 T [151]. (**c**) Schematic representation of the formation of magnetic epithelial cilia. (**d**,**e**) Self-assembly of magnetic nanoparticles in a magnetic field gradient inside a polymer matrix, and optical photos of fabricated cilia under applied external magnetic field [153,154]. Copyright 2010, American Chemical Society. Reproduced from [153]. Copyright 2012, American Chemical Society. Reproduced from [154]. CC BY 3.0.

Cilia function can generally be divided into two categories (Figure 14a):

1. Moving cilia, which produce movement as they permanently pulsate in a certain direction;
2. Non-moving cilia, which usually play the role of sensitive organelles.

Artificial cilia make up a special case of biomimetic microrobots that are either attached to a surface or part of the propulsion system. There are several studies in which microcilia serve as an effective biomedical therapeutic cargo delivery tool. Moreover, they can recover a lost motor function of the surface of mucous membranes and organs. As aforementioned robots, cilia may deliver drugs and perform object manipulation in vivo. An additional advantage of this bioinspired robot type is the ability to deliver liquid drugs to hard-to-reach places due to wireless microfluidic pumping [148,149] and provide a uniform mixing in the case of the simultaneous administration of several types of drugs [150]. Thus, artificial cilia are a promising platform for self-cleaning and droplet manipulation tasks inside cavities, mucous membranes, and body channels.

Some research groups have developed methods for forming elastic and movable artificial magnetic cilia [151,152,153] (Figure 14). Under the influence of an external magnetic field, cilia can move directionally and continuously, imitating biological movements.

Several studies have been devoted to cilia-like arrays made of elastic PDMS containing magnetic cobalt nanoparticles [151,152]. These objects were fabricated by molding the material in a template with microholes (Figure 14b,c). A composite with ordered magnetic chains was created by placing a neodymium magnet under the template during the solidification process. Ben et al. [151] discussed the mechanisms of transporting solid microparticles using magnetic columnar and conical cilia in detail (Figure 14b).

To move polystyrene microspheres in the experiment, a cylindrical permanent magnet NdFeB (B ≈ 0.7 T) mounted on a manipulator was placed above the sample. It was found that the effectiveness of the transportation of viscoelastic microspheres significantly depended on the beat frequency and shape of the flexible cilia. Compared with flexible columnar cilia, conical cilia have demonstrated a higher efficiency in transporting microspheres under the same conditions. It was shown that the greatest transport speed of polystyrene microspheres with conical cilia reached 0.09 mm/s at a beat frequency of 1.7 s per circle.

Another cost-effective approach for the fabrication of artificial cilia by the spontaneous self-assembly of magnetic nanoparticles in chains was proposed in [153,154]. Schematically, the self-assembly process of hair-like structures from microsized beads coated with a soft polymer is shown in Figure 14e. An artificial cilia array was prepared from magnetic microparticles (d = 2.7 µm, Dynabeads^®^ Carboxylic Acid, Waltham, MA, USA) arranged in chains in an external magnetic field of 4–8 mT, perpendicular to the substrate. The polymer coating was formed by polybutylacrylate nanoparticles, which were held on the surface of magnetic beads by electrostatic forces. The artificial cilia were then set in motion in a fluid with an external magnetic field of 5 Hz. The movement was achieved as follows. Four horizontal side poles of an electromagnet were fed with a sinusoidal signal with a 90° phase shift between adjacent poles while maintaining a static vertical field. As a result, the nonreciprocal movement of cilia in a magnetic field leads to a fluid flow rate of 3 µm/s.

A similar growth approach for highly elastic artificial cilia of millimeter length (Figure 14d) from Co nanoparticles (d~800 nm, OM Group, Kokkola, Finland) was proposed in [153]. The length of these cilia was up to three orders of magnitude greater than that of biological cilia.

**Figure 15:**
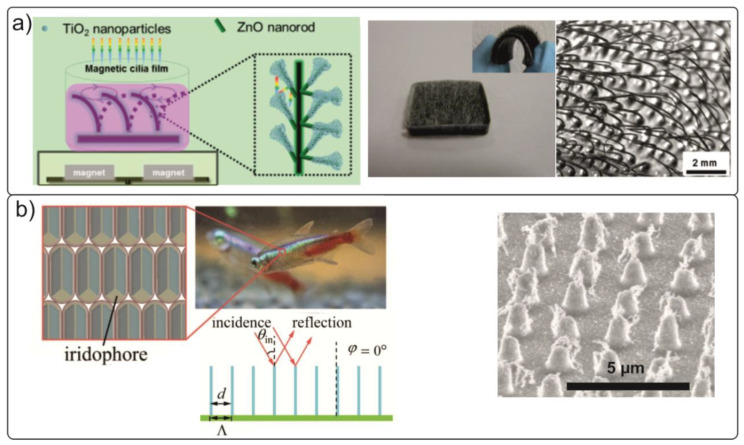
(**a**) Schematic and photo of ZnO/TiO_2_ ciliated films (a bent specimen is shown in the inset). The photo on the right shows the bending of ZnO/TiO_2_ cilia under the action of an external magnetic field [155]. (**b**) Schematic representation of a neon tetra fish and SEM photo of the artificial photonic structure fabricated in [156]. Copyright 2012, American Chemical Society.

Peng et al. [155] proposed and studied a unique multifunctional moving film that incorporates three functional components: magnetic cilia, arrays of ZnO nanorods, and TiO_2_ nanoparticles. Polymer cilia containing magnetic cobalt nanoparticles sized 100–200 nm mimicked natural movements in the magnetic field (Figure 15a). The photocatalytic structure of ZnO/TiO_2_ reduced charge carrier recombination at the heterojunction, imitating the natural photosynthesis. In addition, ZnO nanorods were employed in the active photocatalytic system because the microstructure could improve light absorption owing to multiple reflections. This work demonstrated the high efficiency of photocatalysis in a motile film owing to the functional structures integrated in it.

An interesting way towards dynamic color control using a magnetic field was proposed by Luo et al. [156], who were inspired by neon tetra freshwater tropical fish (Figure 15c). The tilt of the magnetic nanopillars on the substrate under an applied external magnetic field induced a color change in the sample.

This microscopic effect emulates “Venetian blinds” and leads to dynamic changes in the optical properties of the investigated structures in real time. In the field of designing structurally defined colors, considerable success has been achieved owing to the use of bioinspired structures, which are based on the phenomenon of Bragg diffraction [157,158,159,160]. In addition, a number of biomimetic non-magnetic photonic structures have been created, including artificial opals and lamellar interference structures of the butterfly-wing type [161,162,163,164]. The formation of magnetically controlled photonic structures of the neon tetra fish types included the following steps [156]. Iron oxide nanoparticles were collected in nanopillaries in a magnetic field using a special template. The mixture of magnetite (Fe_3_O_4_) nanoparticles (7–10 nm in diameter) with PDMS was named by the authors as ferrofluid polydimethylsiloxane (FFPDMS). Using a periodic array of movable magnetic nanopillars, a spectral shift in the peak due to reflected light from *λ*1 = 528 nm to *λ*2 = 720 nm (Δ*λ* = 192 nm) was demonstrated. A switching time as short as τ = 0.3 s was reached.

A large number of works were devoted to the problem associated with the design of sensing biomimetic ciliated structures [165]. Previously, artificial polymeric cilia on the surface of magnetic sensors were presented [166]. However, the proposed sensor design was subject to the corrosion of its magnetic part (Co_50_Fe_50_). In addition, the toxicity of the cobalt component, as part of the formed cilia, considerably restricts the area of potential application of this design.

**Figure 16:**
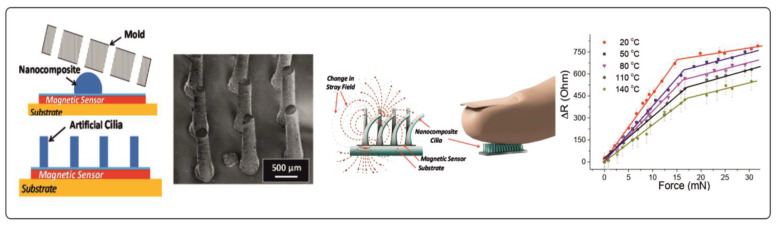
An illustration of the fabrication process of the magnetic cilia tactile sensor, and an SEM image of the sensor. The response of the tactile sensor is initiated by applying vertical pressure in the temperature range from 20 to 140 °C. Reproduced from [168]. CC BY 4.0.

Later, in a number of works, considerable progress was achieved in the creation of artificial ciliated sensors. Alfadhel et al. [167,168] proposed the concept of a biomimetic tactile sensor prepared from a hair-shaped nanocomposite material based on Fe nanowires embedded in a polymer matrix (Figure 16). Such a nanocomposite material is very flexible and possesses high remanent magnetization. When cilia come in mutual contact with a surface, they are deflected and take the shape of the surface. This, in turn, leads to a change in the signal of a giant magnetoresistance (GMR) sensor. The designed flexible structure in combination with the GMR sensor has a higher sensitivity (46 Ohm/mN) than traditional pressure sensors (Figure 16). The proposed magnetoresistive cilia sensor is suitable for the in-flow detection of magnetic particles and is capable of functioning under harsh environmental conditions.

## 7. Conclusions and Prospects for the Future

Composite polymer materials containing additives in the form of magnetic particles are widely used in the development of biomimetic flexible magnetic micro- and nanostructures. In many cases, soft and easily deformable structures ensure increased maneuverability and flexibility under rapidly changing conditions within living organisms. Another widespread option is the deposition of magnetic films onto non-magnetic flexible or rigid templates formed using modern three-dimensional microstructuring methods or additive technologies. Artificially created micro-/nanorobots must imitate the movement of their biological counterparts (microorganisms, cilia, fins, legs, etc.) and implement specific functionality. Such biomimetic structures can be efficiently remotely controlled by weak rotating or oscillating magnetic fields which are harmless to living organisms.

In a number of reported studies, it was shown that artificially created biomimetic micro- and nanoswimmers demonstrate highly efficient movement in viscous liquids. These multifunctional micro- and nanorobots have unique capabilities, including fast movement in complex physiological environments and high transport loads. This allows the use of magnetic micro- and nanoscale biomimetic structures in medicine for the diagnosis and treatment of various diseases, targeted drug delivery within living organisms, and minimally invasive surgery [169,170,171,172,173]. In nature, the limbs of living organisms and the surfaces of many organs and tissues are covered with different ciliated microstructures. The variety of developed physical principles and designs has led researchers to the creation of biomimetic mobile ciliated structures, as well as to the formation of specialized sensory surfaces, i.e., tactile sensors. It is possible to predict the emergence of artificial organ technologies (bronchi, bowel, etc.) containing the functional structures of mobile biomimetic cilia for self-cleaning and droplet manipulation.

The further development of magnetic robotics requires a decrease in the size and an increase in the speed of artificially created bioinspired counterparts for gene delivery and therapy [174,175,176]. Modern control methods should ensure the imitation of the group behavior of micro-/nanorobots with the «intelligence of a bee swarm» using high-precision magnetic fields [177,178,179]. We can expect the development of this topic due to the emergence of more advanced techniques of additive manufacturing, which allows the formation of sophisticated structures on an industrial scale by parallel methods [180,181]. An important aspect for subsequent clinical applications is the development of contemporary biocompatible and non-toxic composite magnetic materials described in the present review. The practical use of such structures in medicine requires a further search for safe materials, including biodegradable ones, which will disappear after the completion of assigned tasks.

The further development of microrobotics can be represented in the following two ways. In the first case, advanced multifunctional robots will solve complex tasks. In the near future, we expect a breakthrough in the fabrication of helical microbots owing to the design of hybrid systems that combine two or more control methods, including magnetic driving and shape memory actuation. In the second case, highly specialized robots will operate as a part of smart groups, where each type will perform its own narrow task. A promising material for the manufacture of microrobots are shape memory alloys that can reversibly deform when the temperature changes. In the future, it is possible to use multifunctional microrobots that perform both delivery and surgery at the same time. Fueled by future size reduction, helical swimmers can be an effective tool for cellular microsurgery, and they may also drill tumors, penetrate cell membranes, and deliver active substances to their nucleus. This will allow operations to be conducted in difficult-to-reach anatomical spaces, small vessels, and brains.

## Figures and Tables

**Table 1:** Comparison of biological microorganisms and artificial bioinspired magnetic robots.

Type	Body Length, μm	Maximum Speed, μm/s	Non-Dimensional Speed, Body Lengths per Second	Reference
Biological Micromotors				
Bovine sperm	30 ÷ 80	10 ÷ 70	~1	-
Flagellate bacteria	3 ÷ 15	20 ÷ 200	~20	-
Infusoria slipper	100 ÷ 300	2000	7 ÷ 20	-
Magnetic Microrobots and Micromotors				
Single-link flexible swimmer	322	158	0.5	Khalil et al. (2014) [74]
Fish-like microrobot	90	220	2.4	Jiang et al.(2021) [75]
Multilink flexible swimmer	15.5	14	0.9	Jang et al. (2015) [76]
Multilink-eel-like swimmer	120	25	0.2	Liao et al. (2019) [77]
Annelid-worm-like microswimmer	20	100	5	Liu et al. (2018) [78]
Helical nanoswimmer	3	15	5	Li et al. (2014) [79]
Biodegradable microswimmer	20	3.5	0.2	Ceylan et al. (2019) [80]
Degradable hyperthermia microrobot	120	114	1	Palagi et al. (2019) [73]
Rigid helical swimmer	38	1.8	0.05	Zhang et al. (2009) [81]
Ciliated microrobot	220	340	1.6	Kim et al. (2016) [82]
Magnetic microparticle chains	57	18	0.3	Cheang et al. (2016) [83]
Magnetic nanoparticle chains	2.8	9.8	3.5	Cheang et al. (2015) [62]
Cube-shaped microrobot	2	20.8	10.4	Chen et al. (2021) [84]
Janus microdimers	10	133	13.3	Yu et al. (2019) [85]
